# A Mistake

**Published:** 1896-10

**Authors:** 


### A Mistake.

A case of an unusual kind came under the notice of the writer recently.

Miss F——, about twenty-one years of age, presented her teeth for inspection. The teeth were in good condition and the mouth healthy; a few small cavities had been filled; the teeth were free from irregularity. The mouth being opened, it was at once noticeable that the left superior cuspid was absent, and a space sufficient for its accommodation remained between the lateral incisor and first bicuspid.

The absence of the tooth caused a very pronounced depression of the surface of the part in which the tooth had stood, and not only this, but the lip and face covering this part was quite depressed, so much so as to mar the symmetry of the face, especially to a close observer. Now for the history:

At the age of about fifteen this tooth was not fully grown, having been a little tardy in its eruption. The young lady, at about the time above indicated, having her teeth examined, called the attention of her dentist to this tooth, when he promptly replied: "Oh, that is a temporary tooth and must be removed to give place to the permanent one." The patient remarked that she "supposed she had shed all her temporary teeth." To which the dentist replied: "No, it is a temporary tooth and must be removed; you go to Dr. ——, take gas, and have it extracted." And at once she obeyed, but to her life-long regret.

Now, what shall be said about this case? Well, the dentist is not a quack; he is not ignorant; has had good educational advantages for his profession; he has good a practice, that ought to satisfy any one. Now, why this blunder? Simply inexcusable carelessness, that will probably be remembered against him through life, and should bring upon him the sharpest censure. This case emphasizes the importance of the utmost care in all examinations of the teeth.

Of the extractor who removed this tooth we know nothing, except what is revealed by this operation. He seems to have been little, if anything, more than a machine, ready to obey any request regardless of consequences, even to the removal of a sound, healthy, cuspid tooth, which will impair the symmetry of a beautiful oval of teeth for life.

If so gross a mistake occurs in so simple a case as this, what may not happen in the more complicated and abstruse cases?

